# T-DNA alleles of the receptor kinase THESEUS1 with opposing effects on cell wall integrity signaling

**DOI:** 10.1093/jxb/erx263

**Published:** 2017-08-01

**Authors:** David Merz, Julia Richter, Martine Gonneau, Clara Sanchez-Rodriguez, Tobias Eder, Rodnay Sormani, Marjolaine Martin, Kian Hématy, Herman Höfte, Marie-Theres Hauser

**Affiliations:** 1 Department of Applied Genetics and Cell Biology, University of Natural Resources and Life Sciences, Vienna, Austria; 2 Institut Jean-Pierre Bourgin, INRA, Centre National pour la Recherche Scientifique, AgroParisTech, Université Paris-Saclay, Versailles Cedex, France; 3 Department of Biology, ETH Zürich, 8092 Zürich, Switzerland

**Keywords:** Cell elongation, cellulose synthesis, cell wall integrity signaling, CrRLK1L receptor, gene silencing, isoxaben

## Abstract

Perturbation of cellulose synthesis in plants triggers stress responses, including growth retardation, mediated by the cell wall integrity-sensing receptor-like kinase (RLK) THESEUS1 (THE1). The analysis of two alleles carrying T-DNA insertions at comparable positions has led to conflicting conclusions concerning the impact of THE1 signaling on growth. Here we confirm that, unlike *the1-3* and other *the1* alleles in which cellular responses to genetic or pharmacological inhibition of cellulose synthesis are attenuated, *the1-4* showed enhanced responses, including growth inhibition, ectopic lignification, and stress gene expression. Both *the1-3* and *the1-4* express a transcript encoding a predicted membrane-associated truncated protein lacking the kinase domain. However, *the1-3*, in contrast to *the1-4*, strongly expresses antisense transcripts, which are expected to prevent the expression of the truncated protein as suggested by the genetic interactions between the two alleles. Seedlings overexpressing such a truncated protein react to isoxaben treatment similarly to *the1-4* and the full-length THE overexpressor. We conclude that *the1-4* is a hypermorphic allele; that THE1 signaling upon cell wall damage has a negative impact on cell expansion; and that caution is required when interpreting the phenotypic effects of T-DNA insertions in RLK genes.

## Teaser

Antagonistic growth effects of two T-DNA alleles in THESEUS1 correlate with the expression of a predicted functional THE1 protein lacking the kinase domain and antisense transcripts, respectively.

## Introduction

**Fig. 1.**
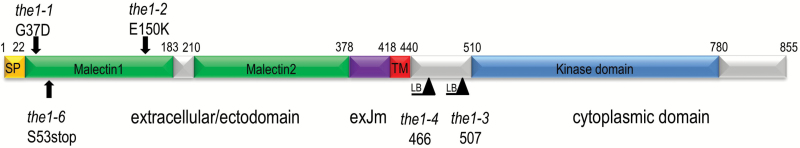
Overview of the domain structure and mutant alleles of THESEUS1. SP, TM, and exJM correspond to signal peptide, transmembrane domain, and extracellular juxtamembrane region, respectively. Numbers indicate amino acid residues. Arrows indicate the position and amino acid changes of the mutant alleles. LB indicates the left border of the T-DNA insertion. G, D, E, K, and S denote glycine, aspartic acid, glutamic acid, lysine, and serine, respectively.

The inhibition of cellulose synthesis in plants causes stress responses, including growth retardation. The latter is not only the result of the structural changes in the cell wall linked to a reduced cellulose content but involves active growth inhibition mediated by a receptor kinase that appears to act as a wall integrity sensor (Hématy *et al.*, 2007). This receptor kinase was identified in a screen for suppressors of the short hypocotyl and ectopic lignin phenotype of the cellulose synthase mutant *cesa6*^*prc1-1*^. Three mutant alleles were identified in the *THESEUS1* (*THE1*) gene, which all partially suppressed this phenotype in *cesa6*^*prc1-1*^ and other cellulose-deficient mutants. Alleles *the1-1* and *the1-2* carry amino acid changes in the predicted ectodomain. This domain comprises two copies of a malectin-like domain, which in animal cells binds to carbohydrate epitopes and also in plants may bind to cell wall carbohydrates (Schallus *et al.*, 2008; Nissen *et al.*, 2016; Voxeur and Höfte, 2016). Allele *the1-3* carries a T-DNA insertion behind the cytosolic and transmembrane domain-encoding portion of the intron-less gene (Fig. 1). The suppressor phenotype of the three independent alleles suggested that THE1 negatively regulates growth as part of a more general stress response including the accumulation of reactive oxygen species (ROS) and ectopic lignin (Denness *et al.*, 2011), and the up-regulation of jasmonic acid (JA)-regulated genes. The latter include genes involved in ROS detoxification and the synthesis of defense compounds such as indole-glucosinolates (Hématy *et al.*, 2007).

*THE1* belongs to the 17-member *Catharanthus roseus* receptor-like kinase 1-like (CrRLK1L) gene family (Li *et al.*, 2016; Nissen *et al.*, 2016), the most intensively studied member of which is *FERONIA* (*FER*). FER has been implicated in numerous biological processes, such as mechanosensing (Shih *et al.*, 2014), pollen tube recognition at the micropyle (Escobar-Restrepo *et al.*, 2007), bacterial pathogen interaction (Keinath *et al.*, 2010), brassinosteroid responsiveness (Deslauriers and Larsen, 2010), and root hair development (Duan *et al.*, 2010). In addition, FER is a receptor for secreted peptides of the RALF (rapid alkalinization factor) family (Haruta *et al.*, 2014; Stegmann *et al.*, 2017), which trigger alkalinization of the cell surface and inhibition of cell elongation (Haruta *et al.*, 2014).

Roles in cell elongation have also been demonstrated for other family members *CURVY* (*CVY1*), *HERKULES1* (*HERK*), *HERK2*, and *ANXUR1* and *2* (*ANX1/2*). *CVY1* regulates trichome and pavement cell morphogenesis and *cvy1* mutants develop faster and have a higher fecundity (Gachomo *et al.*, 2014). Double mutants of *ANX1/2* are required to maintain elongation in pollen tubes (Boisson-Dernier *et al.*, 2009; Miyazaki *et al.*, 2009) while overexpression of *ANX1/2* inhibits pollen tube elongation (Boisson-Dernier *et al.*, 2013). Mutants for *HERK1* or *2* did not show growth defects, but developed, in combination with the *THE1* mutant allele *the1-4*, smaller leaves and petioles. This suggested a partially redundant growth-promoting role for HERK1, HERK2, and THE1 even in the absence of cell wall damage (Guo *et al.*, 2009*a*, *b*). The *the1-4* allele in combination with a *AtKINESIN-13A* mutant triggered a similar reduction of cell expansion in petals (Fujikura *et al.*, 2014). These findings were contradictory to the growth-inhibiting rather than the growth-promoting role for THE1 inferred from the *cesa6*^*prc1-1*^ suppressor mutants (Hématy *et al.*, 2007).

Here we show that *the1-4*, in contrast to the other four alleles, enhances rather than suppresses the growth defects of *cesa6*^*prc1-1*^ and *cesa3*^*je5*^, and thus mimics the effect of THE1 overexpression. We next demonstrate that *the1-4* is a hypermorphic gain-of-function allele, which expresses a transcript encoding a predicted membrane-associated truncated protein, lacking the kinase domain. This truncated protein appears to trigger a stress response more efficiently than the wild-type protein upon inhibition of cellulose synthesis, perhaps through the interaction with other membrane receptor kinases. In contrast, *the1-3*, which carries a T-DNA insertion at a position comparable with that of *the1-4*, strongly expresses antisense transcripts, which presumably interfere with the expression of the truncated THE1 protein from the sense transcripts and hence can be considered a partial or complete loss-of-function allele like *the1-1*, *the1-2*, and the new knock out allele *the1-6*. These observations confirm that THE1 signaling negatively, not positively, affects cell expansion upon inhibition of cellulose synthesis. The availability of both loss-of-function and gain-of-function alleles will be useful for the study of cell wall integrity signaling in Arabidopsis and, more generally, underscore that caution is required when interpreting the phenotypic effects of RLK and T-DNA insertion mutants.

## Materials and methods

### Plant materials

The *the1-1* (G37D) and *the1-2* (E150K) mutants were discovered in a suppressor screen with the cellulose-deficient mutant *cesa6*^*prc1-1*^ (Hématy *et al.*, 2007) and the novel ethyl methanesulfonate (EMS)-induced allele *the1-6* (S53Stop) in a suppressor screen of *ctl1-1/pom1*. *the1-3* (FLAG_201C06) and *the1-4* (SAIL_683_H03) are both T-DNA mutants in either the Wassilewskija (WS-0) or Columbia (Col-0) background (Hématy *et al.*, 2007; Guo *et al.*, 2009*a*). Both T-DNA lines were crossed into the cellulose-deficient *CELLULOSE SYNTHASE A6* and *A3* mutants: *cesa6*^*prc1-1*^, *cesa6*^*prc1-8*^, *cesa3*^*je5*^, and *cesa3*^*eli1-1*^. Homozygous plants were selected after PCR genotyping and through sequencing of the *cesa3*^*je5*^ gene.

An overexpressing green fluorescent protein (GFP) reporter fusion (THE1:GFP) controlled by a double *Cauliflower mosaic virus* (CaMV) 35S promoter in the Col-0 accession (Hématy *et al.*, 2007) was crossed with *cesa3*^*je5*^ and with double mutants of *cesa3*^*je5*^*the1-3* and *cesa3*^*je5*^*the1-4*. Homozygous F_3_ generations were selected based upon their growth phenotype and PCR genotyping.

### Generating ECD–TM^THE1^–YFP-overexpressing plants

A 1510 bp fragment of *THE1* coding for the extracellular domain (ECD), the transmembrane (TM) domain, and the juxtamembrane sequence up to the kinase domain (amino acid 510) was PCR amplified with primers THE1-Start and TM-JTM-Lo, and cloned into the reconstituted *Sma*I site of pSmile-YFP. pSmile-YFP was generated by inserting the yellow fluorescent protein (YFP) coding sequence (CDS) into the *Sma*I site of the pBIB-Hygro-derived plasmid pMagic (Nesi *et al.*, 2002) containing a double CaMV 35S promoter. Wild-type *Arabidopsis thaliana* accession Col-0 was transformed with the *Agrobacterium tumefaciens* strain C58 pMP90 with the floral-dip method (Clough and Bent, 1998). Transformants were selected on Murashige and Skoog (MS) medium containing 50 mg l^–1^ hygromycin (Duchefa, France).

### Growth conditions

Seeds were surface-sterilized and placed on sterile MS agar plates supplemented with 4.5% sucrose (w/v) as described previously (Hauser *et al.*, 1995). After 2 d (1 week for the WS background) of imbibition at 4 °C in darkness, the plates were placed in a 22 °C growth chamber with constant white light (80 µmol m^–2^ s^–1^).

For the production of etiolated seedlings, seeds were sterilized and placed on MS plates without sucrose or supplemented with 2.5% sucrose. After imbibition and before wrapping the plates in aluminum foil, they were exposed for 4–5 h to white light and subsequently incubated in a vertical position at 22 °C.

Isoxaben treatment was done with 4-day-old etiolated seedlings by cultivating them on a 75 µm nylon mesh for easy transfer to MS medium containing 100 nM isoxaben or the same amount of methanol as solvent control. After 6 h further growth in the dark, the seedlings were harvested, shock frozen in liquid nitrogen, and RNA was isolated for expression analyses.

Plant growth on soil (1:1 mix of perlite and soil) was initiated from *in vitro* cultured, 10- to 14-day-old seedlings. Rosette and inflorescence phenotypes were photographed after 4 or 5 weeks, respectively.

### Growth analyses and lignin staining

Hypocotyl lengths were measured from pictures of either 5- or 7-day-old etiolated plants of *the1* mutants in the *cesa6*^*prc1-1*^ and *cesa3*^*je5*^ background, respectively, with the ImageJ freehand tracking and the measuring tool from photographs.

For lignin staining, seedlings were fixed and cleared in methanol/acetate (3:1) for 1 h, washed with water, stained with 1% phloroglucinol in 6 N HCl/46% ethanol at room temperature for 15 min, and mounted in either water or chloralhydrate solution (16 g of chloralhydrate dissolved in 5 ml of phosphate buffer with 17.5% glycerol).

### Expression analyses by semi-quantitative and quantitative real-time PCR

Total RNA was isolated with TRI REAGENT (MRC, Cincinnati) according to the manufacturers’ protocols. First-strand cDNA was synthesized from 2.5 µg of RNA with the Superscript III Moloney murine leukemia virus (MMLV) reverse transcriptase (Invitrogen) after DNase I digestion (Roche) as described by Karsai *et al.* (2002). For sense cDNA synthesis, oligo(dT)_18_ primer, and for antisense cDNA synthesis, the gene-specific primer *THE1_midF* or the T-DNA-specific primers *LB3* and *LB4*, were used (Supplementary Table S1 at *JXB* online).

For semi-quantitative determination of sense and antisense expression primers downstream (*THE1_3endF/THE1_endR*) and upstream (*THE1_5endF/THE1_5endR*), the T-DNA insertions were used. For antisense fragments originating from the T-DNA of *the1-3*, the primers *THE1_midF* and *LB4* were used and for those originating from the T-DNA of *the1-4* the primers *THE1_midF* and *LB3* were used and compared with the expression of the housekeeping gene *BETA-TUBULIN9* (*TUB9*). Duplicate PCRs from 3–4 independent cDNAs (biological replicates) were evaluated and shown by representative, ethidium bromide-stained gel images.

Reverse transcription–quantative PCR (RT–qPCR) expression analyses were carried out using the Hot FirePol EvaGreen qPCR Mastermix (Solis Biodyne) with the Rotorgene 3000 (Qiagen). Primers for *THE1* expression analyses were the same as for the semi-quantitative approach. Primers of the THE1-dependent genes, 5g19110/EDGP and 2g26530/AR781, and the reference gene *UBIQUTIN EXTENSION PROTEIN 5* (*UBQ*) are listed in Supplementary Table S1. Absolute and relative expression was calculated with a dilution series of purified PCR fragments of known molar concentrations in each RT–qPCR run. Each sample was measured in triplicate and from 3–4 independent cDNAs. The identities of amplicons were verified with melting curve analyses.

## Results

### THE1 *alleles have opposing effects on plant growth, ectopic lignification, and gene expression*

**Fig. 2.**
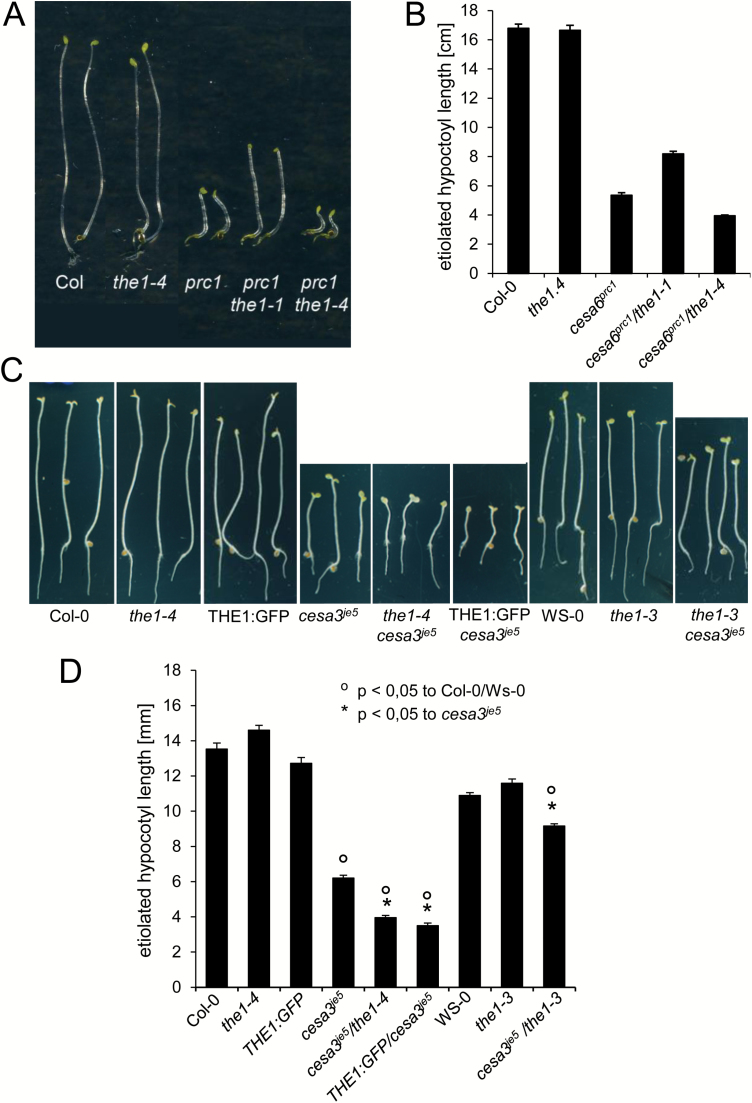
Seedling development of loss- and gain-of-function alleles of *THE1* in combination with cellulose-deficient mutants. (A) Five-day-old dark-grown seedlings of the wild type (Col-0), *the1-4*, *prc1*, and the double mutants *prc1/the1-1* and *prc1/the1-4*. (B) Hypocotyl length quantification of etiolated seedlings in (A). (C) Seven-day-old etiolated seedlings of the wild type (Col-0, WS), *the1-4*, THE1:GFP, *cesa3*^*je5*^, *the1-3*, and the double mutants *the1-4/cesa3*^*je5*^, THE1:GFP*/cesa3*^*je5*^, and *the1-3/cesa3*^*je5*^. (D) The quantification of hypocotyl length of (C). The graphs show the average ±SE of 37–40 seedlings in (B) and 27–152 seedlings in (D). * and °*P*<0.05 according to Student’s *t*-test compared with the wild type or *cesa3*^*je5*^. (This figure is available in colour at *JXB* online.)

We studied four previously described *THE1* alleles (*the1-1* to *the1-4*) and a novel allele (*the1-6*) isolated from a suppressor screen of the cellulose-deficient mutant *ctl1-1/pom1*. *the1-6* carries a point mutation causing a (TCA to TAA) change of S53 into a stop codon (Fig. 1). This truncates the protein within the first malectin domain and hence can be considered as a complete loss-of-function allele. The *the1-6* allele was separated from the *ctl1-1* mutation before further analysis. The positions of the respective mutations in the *THE1* sequence are summarized in Fig. 1. We showed previously that *the1-1*, *the1-2*, and *the1-3* all partially suppress the dark-grown hypocotyl growth defect of the *cesa6*^*prc1-1*^ mutant (Hématy *et al.*, 2007). Interestingly, the *the1-4* allele had the opposite effect: it enhanced the phenotype of *cesa6*^*prc1-1*^, while it did not show a hypocotyl growth phenotype in a wild-type background (Fig. 2A, B). To confirm these observations for another cellulose-deficient mutant, we combined *the1-3*, *the1-4*, and a 35S::THE1:GFP overexpression line (named THE1:GFP), respectively, with the weak allele of *CELLULOSE SYNTHASEA3*, *cesa3*^*je5*^. This allele is identical to *multiple response expansion1* (*mre1*) (Pysh *et al.*, 2012) and carries a point mutation at position 916 causing an amino acid change from a conserved glycine to glutamate. Based on *in silico* analysis, this mutation causes a shift in the location of transmembrane domain (TMD) 5 from amino acids 913–929 in the wild type to amino acids 919–935 in *cesa3*^*je5*^. This shift leads to an enlargement of the cytoplasmic loop between TMD 4 and 5 and a reduction of the cell wall-facing loop between TMD 5 and 6 of six amino acids. Another possibility is that TMD 5 is a cytosolic interfacial helix similar to the bacterial catalytic unit of cellulose synthase (BcsA) and involved in the regulation of UDP-glucose access to the nearby catalytic pocket (Slabaugh *et al.*, 2014). This scenario and topological change, however, are incompatible with the recent finding of cysteine acylation at the C-terminus (Kumar *et al.*, 2016) unless TMD 6 behaves as a re-entrant segment that enters the membrane but does not cross it. Again, the hypocotyl growth defect of *cesa3*^*je5*^ was suppressed in *the1-3* and enhanced in *the1-4* and THE1:GFP, whereas neither *the1-3*, *the1-4*, nor THE1:GFP showed a detectable dark-grown phenotype in a wild-type background (Fig. 2C, D). Similar observations were made for seedlings grown in the light on 4.5% sucrose, a condition that enhances growth phenotypes of many cell wall mutants (Hauser *et al.*, 1995). While *cesa3*^*je5*^ seedlings develop short radially swollen roots, they are even shorter in *the1-4*/*cesa3*^*je5*^ and *cesa3*^*je5*^/THE1:GFP, and longer and less swollen in *the1-3*/*cesa3*^*je5*^ double mutants (Fig. 7B; Supplementary Fig. S1). It has been shown previously that ectopic lignin accumulation in *cesa6*^*prc1-1*^ and *cesa3*^*eli1-1*^ depends on functional THE1 (Hématy *et al.*, 2007). Also *cesa3*^*je5*^ accumulates ectopic lignin in etiolated hypocotyls. While this phenotype is reduced in *cesa3*^*je5*^/*the1-3*, it is strongly enhanced in *cesa3*^*je5*^/*the1-4* and *cesa3*^*je5*^/THE1:GFP (Supplementary Fig. S2).

**Fig. 3.**
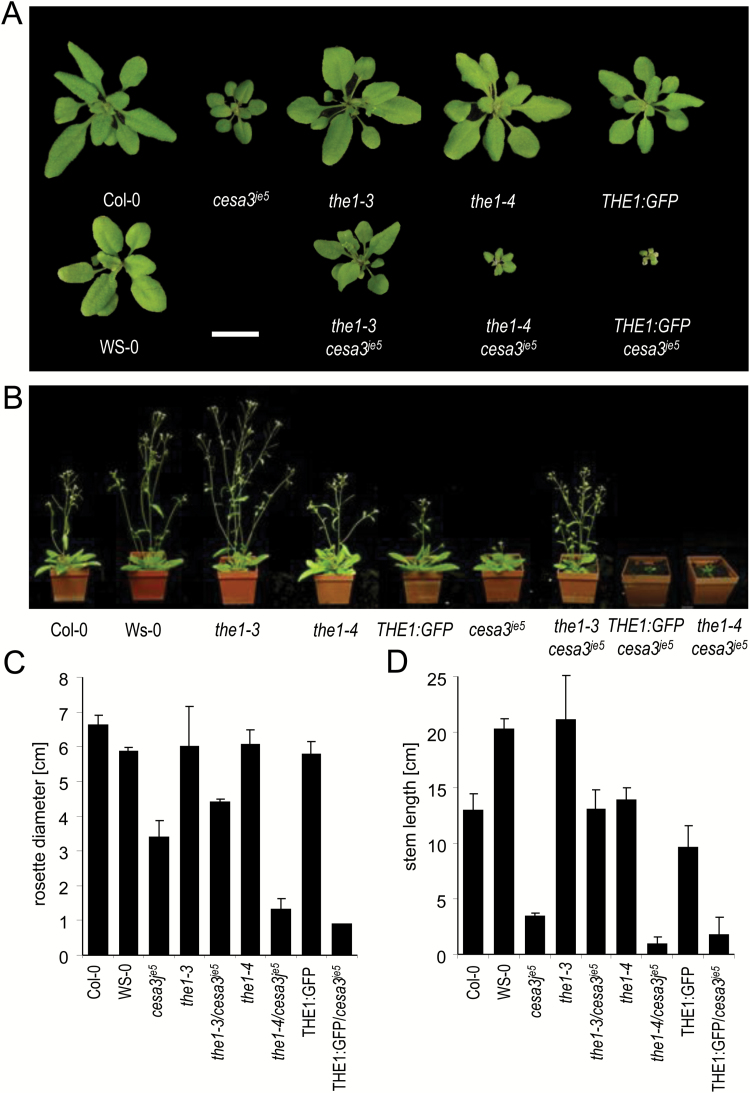
Phenotypes of loss- and gain-of-function alleles in combination with cellulose-deficient mutants on soil. (A) Rosette phenotypes of 4-week-old plants of the wild type (Col-0, WS), *the1-4*, THE1:GFP, *cesa3*^*je5*^, *the1-3*, and the double mutants *the1-4/cesa3*^*je5*^, THE1:GFP/*cesa3*^*je5*^, and *the1-3/cesa3*^*je5*^, (B) The same genotypes 2 weeks later. (C) Quantification of the rosette diameters and (D) the stem lengths. The graphs show the average ±SE for at least four plants.

The opposite effects of *the1-3* and *the1-4* alleles were also observed in soil-grown adult plants of *cesa3*^*je5*^ (Fig. 3; Supplementary Fig. S3). The rosette diameter was not significantly different among wild types, *the1-3*, *the1-4*, and THE1:GFP, while *cesa3*^*je5*^ showed a significantly smaller rosette diameter (Fig. 3A, C). This phenotype was also reverted in *cesa3*^*je5*^/*the1-3* and enhanced in *cesa3*^*je5*^/*the1-4* or *cesa3*^*je5*^/THE1:GFP. Plant heights followed the same trend of opposite effects, with *cesa3*^*je5*^/*the1-4* or *cesa3*^*je5*^/THE1:GFP hardly surviving on soil (Fig 3B, D; Supplementary Fig. S3). In conclusion, alleles *the1-1*, *the1-2*, and *the1-3* partially or completely suppressed the dark-grown hypocotyl phenotypes of *cesa6*^*prc1-1*^ or *cesa3*^*je5*^ as well as the adult plant phenotypes of *cesa3*^*je5*^ and *cesa3*^*eli1-1*^, whereas *the1-4*, like THE1:GFP, enhanced these phenotypes.

**Fig. 4.**
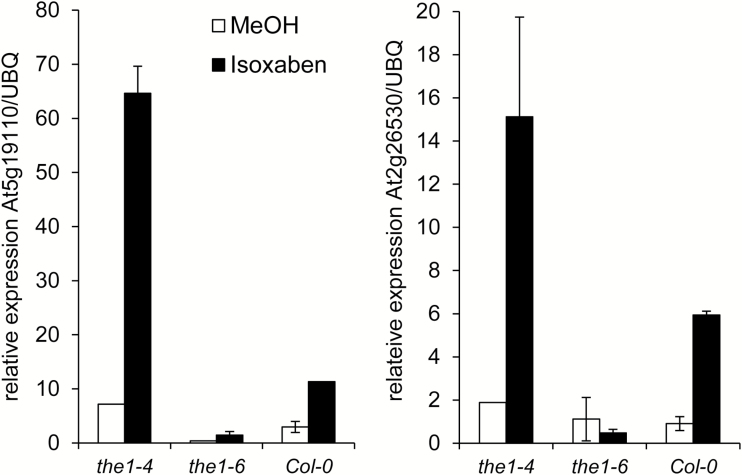
Opposite effects of loss- and gain-of-function alleles of *THE1* on gene expression. Relative expression of THE1-dependent genes At5g19110 and At2g26530 normalized with the reference gene UBQ5 of etiolated seedlings after 6 h of isoxaben or mock treatment in the dark. The graph represents the mean ±SE of three biological replicates with each of three technical replicates.

To avoid long-term pleiotropic effects typically observed in cellulose-deficient mutants, we used the cellulose synthesis inhibitor isoxaben to challenge the THE1 pathway in a more controlled way (Heim *et al.*, 1989; Scheible *et al.*, 2001; Desprez *et al.*, 2002, 2007; Wormit *et al.*, 2012). As a read out, we quantified the transcript levels of two genes that previously had been shown to be up-regulated in *cesa6*^*prc1-1*^ but not in *cesa6*^*prc1-1*^*/the1-1.* As expected, 6 h isoxaben treatment of 4-day-old dark-grown seedlings caused a 3.8-fold and 6.5-fold increase of At5g19110 and At2g26530 transcript levels in wild-type seedlings compared with the solvent control (Fig. 4). Interestingly, in the complete loss-of-function background *the1-6*, the expression induction was abolished, whereas in *the1-4* the induction was strongly enhanced in a way similar to that observed in THE1:GFP (Hématy *et al.*, 2007). Together, these results demonstrate that the *the1-4* allele has an effect on growth and gene expression opposite to that of loss-of-function *the1* alleles.

### the1-3 *and* the1-4 *are loss-of-function and gain-of-function alleles, respectively*

The opposite effect of the two alleles is surprising given the fact that they are both the result of T-DNA insertions at positions very close to each other (amino acids 507 and 466 for *the1-3* and *the1-4*, respectively), corresponding to the cytoplasmic region just downstream of the membrane-spanning domain (Fig. 1). If expressed, the resulting truncated proteins would comprise the N-terminal extracellular domain, the membrane-spanning domain, and a short stretch of amino acids derived from the intracellular domain, but without the kinase domain.

**Fig. 5.**
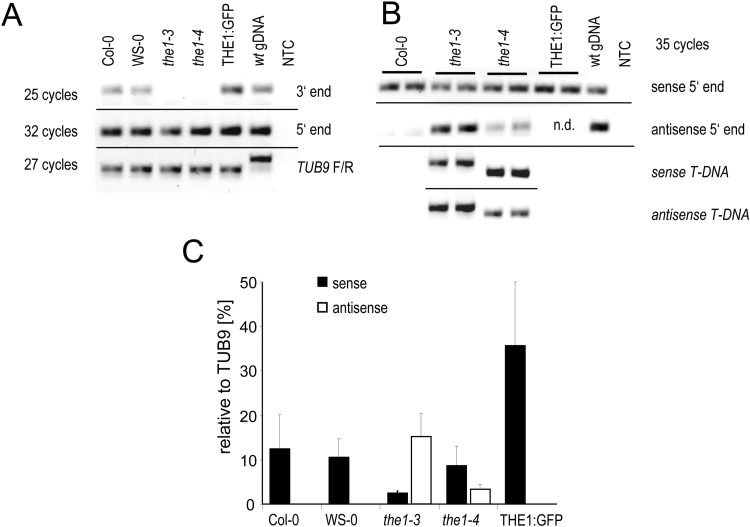
*THE1* sense and antisense expression in *the1-3* and *the1-4*. (A) Semi-quantitative expression of *THE1* 5' and 3' of the T-DNA insertion. (B) Semi-quantitative expression of sense and antisense transcripts of *THE1* 5' of the T-DNA and with one primer located on the T-DNA. Horizontal lines signify images from the same analysis and gel. (C) RT–qPCR expression analyses of the wild type (Col-0, WS), *the1* mutant alleles, and the THE1:GFP overexpressor normalized to the reference gene *TUB9*. The graph represents the mean ±SE of four biological with each of three technical replicates.

To understand the opposing phenotypes, we investigated the transcripts produced at the *THE1* locus in the two mutants. RT–qPCR showed that in both mutants, THE1 transcripts downstream of the T-DNA insertion were absent (Fig. 5A), whereas the transcript levels corresponding to the 5' end of *THE1* were unaltered in the wild type, *the1-3*, and *the1-4* (Fig. 5A). Although the two alleles were produced with different T-DNA vectors [*the1-3*, pGKB5 (Bouchez *et al.*, 1993; Samson *et al.*, 2002); *the1-4*, pDAP101 (Sessions *et al.*, 2002)], both contain promoters facing towards the left border (LB). While in *the1-3* the 35S promoter of the T-DNA was driving a BASTA resistance gene and is ~2000 bp from the LB, in *the1-4*, pDAP101 contains the bidirectional MAS (mannopine synthase) promoter (Velten *et al.*, 1984) 96 bp from the LB. To test the possibility that T-DNA-driven antisense transcripts were present, we quantified these as well. Indeed, in both alleles, we detected antisense transcripts upstream of the T-DNA insertion (Fig. 5B) but *the1-3* was present in significantly higher amounts than *the1-4* (Fig. 5C). The ratio between antisense and sense transcripts was ~7 for *the1-3* and 0.7 for *the1-4* (Fig. 5C).

**Fig. 6.**
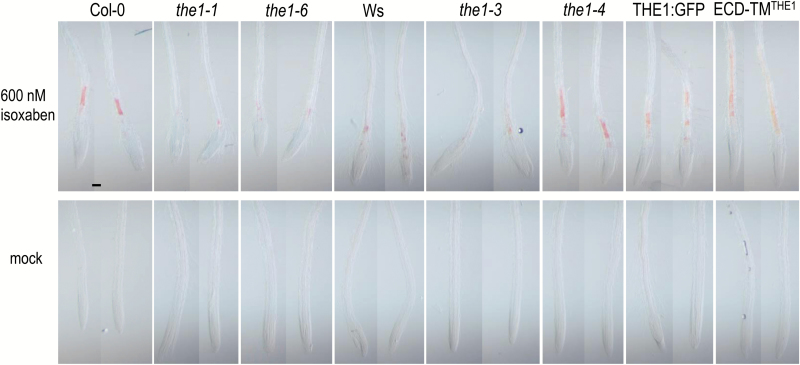
Ectopic lignification of loss- and gain-of-function alleles upon chemical inhibition of cellulose biosynthesis. Shown are roots from 6-day-old seedlings cultivated on half-strength MS with 1% sucrose and incubated for 16 h with 600 nM isoxaben or the mock control. Seedlings were stained with 1% phloroglucinol for 15 min. Pictures were taken immediately after mounting the seedlings in water. The scale bar corresponds to 100 µm.

We hypothesize that *the1-4* sense transcripts produce a truncated protein, whereas in *the1-3*, the accumulation of the encoded protein is prevented by the presence of antisense transcripts, which presumably act post-transcriptionally. Unfortunately, despite repeated attempts, we have not been able to detect such truncated proteins with an antiserum directed against the THE1 ectodomain. To examine this hypothesis, we overexpressed the truncated THE1 protein without the kinase domain (ECD–TM^THE1^) in Col-0 and studied the response to isoxaben. While ectopic lignification in roots of all the knock out mutants, *the1-1*, *the1-3*, and *the1-6* was weaker as in their corresponding wild types, the full-length THE:GFP overexpressor, *the1-4*, and the transgenes overexpressing the truncated ECD–TM^THE1^ accumulated lignin to a much higher extent than the wild type upon isoxaben treatment (Fig. 6). Thus the kinase domain is not required for THE1-induced responses upon cellulose deficiency.

### *Both* the1-3 *and* the1-4 *alleles are semi-dominant*

**Fig. 7.**
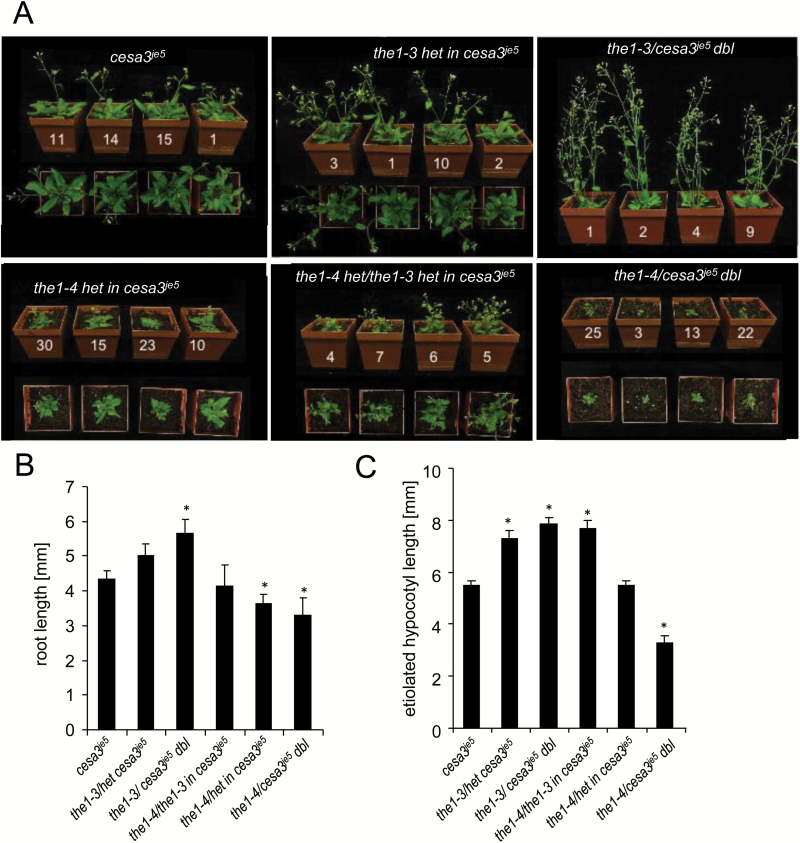
Semi-dominant phenotypes of *the1-3* and *the1-4* in cellulose-deficient backgrounds. (A) Four-week-old plants. All plants are homozygous for *cesa3*^*je5*^ and either wild type, heterozygous for *the1-3* and/or *the1-4*, and homozygous for *the1-3* or *the1-4*. (B) Root length 7 d after germination and (C) etiolated hypocotyl length of 5-day-old seedlings of the above-mentioned genotypes. Graphs represent the mean ±SE of 5–35 seedlings in (B) and 9–24 seedlings in (C). Significant differences from *cesa3*^*je5*^ are indicated according to Student’s *t*-tests with *P<0.05.

According to our hypothesis, both the increased and decreased sensitivity to cell wall perturbation in *the1-4* and *the1-3*, respectively, would involve mechanisms with dominant effects. Indeed, the accumulation of a truncated membrane-bound ectodomain in *the1-4* is also expected to enhance the signaling strength in a heterozygote, whereas the accumulation of an inhibitory antisense transcript in *the1-3* might also have dominant effects, by inhibiting the accumulation of the endogenous THE1 protein. To investigate this, we analyzed the growth phenotype of heterozygotes for one or the other allele in a homozygous *cesa3*^*je5*^ background. As expected, heterozygote seedlings for *the1-3* and *the1-4* developed primary roots and etiolated hypocotyls of a length intermediate between those of *cesa3*^*je5*^ and their homozygous counterparts (Fig. 7B, C). The semi-dominance of the hypocotyl elongation was also seen in the *the1-3*/*cesa6*^*prc1-8*^ combinations (Supplementary Fig. S4). In addition, consistent with our hypothesis, *the1-3* suppressed the enhancing effects of *the1-4* in the transheterozygote.

For soil-grown plants, the intermediate phenotypes of heterozygous rosettes and inflorescences and the epistasis of *the1-3* over *the1-4* were even more evident (Fig. 7A).

## Discussion

Here we showed that different alleles of *THE1* have opposite phenotypic effects: *the1-4* enhances whereas *the1-1*, *the1-2*, *the1-3*, and the novel allele *the1-6* suppress the reduced growth and (at least for *the1-1*, *-2*, and *-3*) ectopic lignification phenotypes in a cellulose-deficient background. In addition, the expression of two genes induced by the cellulose synthesis inhibitor isoxaben was enhanced and suppressed in *the1-4* and *the1-6*, respectively. The same two genes were also up-regulated in a cellulose-deficient *cesa6*^*prc1-1*^ background relative to the wild type, and this effect was further enhanced by THE1:GFP and suppressed by *the1-1* and *the1-3* (Hématy *et al.*, 2007).

**Fig. 8.**
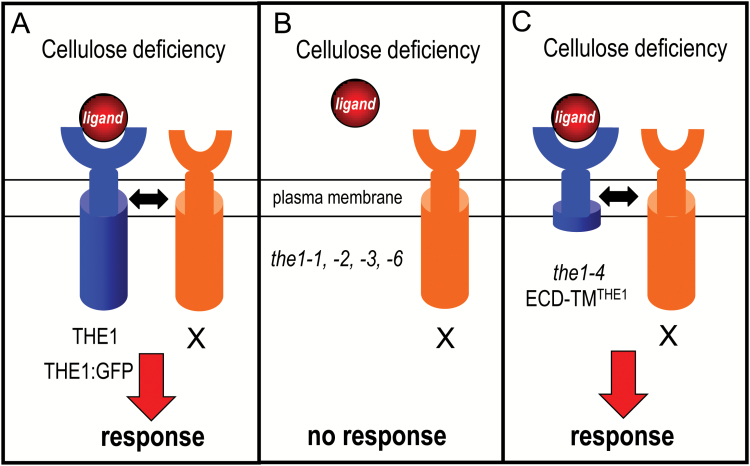
Model for the action of the loss- and gain-of-function alleles of *THESEUS1*. (A) Cellulose deficiency generates a ligand (red) which binds to the extracellular domain and activates THE1 or THE1:GFP (blue). THE1 either signals directly or associates with one or multiple partners (orange). The activation of this pathway suppresses growth and cell elongation and induces transcriptional changes. (B) In loss-of-function plants (*the1-1*, *-2*, *-3*, and *-6*), plants express no or reduced levels of THE1 which cannot trigger growth responses to or sufficiently efficient associations with signal transmitting partners. (C) In gain-of-function plants (*the1-4*), the truncated THE1 protein without the cytoplasmic kinase domain is able to associate with the signal transmitting partners and induce strong growth effects. The truncated *the1-4* and the overexpressed THE1:GFP proteins are less sensitive to feedback inhibition mediated by the cytoplasmic domain and cause semi-dominant phenotypes.

The T-DNA insertions of *the1-3* and *the1-4* are only 120 bp apart in a region between the transmembrane and kinase domain. The enhancing effect of *the1-4* can be explained by the presence of a truncated THE1 protein lacking the kinase domain that is able to trigger cell wall damage-induced stress responses more efficiently than the wild-type protein. The kinase domain therefore is not required for THE1 activation, like for FER, for which a kinase-dead version still complements the defective pollen tube reception phenotype (Kessler *et al.*, 2015). The hypersensitive *the1-4* phenotype suggests that the THE1 kinase domain is required for turning down THE1 activity. RLKs often occur in heteromeric complexes, which can also contain kinase-defective RLKs or receptor-like proteins (RLPs) that influence RLK-mediated signaling. Therefore, it is possible that THE1 can act as co-receptor in such a complex (Castells and Casacuberta, 2007; Gust and Felix, 2014; Liebrand *et al.*, 2014) (Fig. 8). Potential candidates for such signaling partners are other CrRLK1L family members including HERK1/2 or FER, some of which are co-expressed with THE1 (Lindner *et al.*, 2012). The predicted truncated THE1 protein in *the1-4* contains the ectodomain, the TMD, and ~26 amino acids of the cytoplasmic domain. Within the ectodomain, the extracellular juxtamembrane region (exJM) is highly conserved in all CrRLKs. In FER, this region interacts with the glycosylphophatidylinositol (GPI)-anchored co-receptors LORELEI (LRE) and LRE-LIKE (LLG1-3) (Li *et al.*, 2015). Furthermore, TMDs can also serve as interaction platforms (Cymer *et al.*, 2012), for instance through the ‘glycine zipper’ motif in the TMD of the RLK SOBIR1/EVR (SUPPRESSOR OF BIR1-1/EVERSHED), which mediates interaction with several RLPs (Bi *et al.*, 2016). Confirmation of these hypotheses awaits the identification of TMD and/or ectodomain interaction partners of THE1 and functional studies with constitutively kinase-active and kinase-dead versions.

The expression of the 5' end of *THE1* is reduced in *the1-3* and correlates with the presence of an antisense RNA initiated from the T-DNA. Interestingly, both T-DNAs contain promoters at the LB, which, given their orientation, would generate *THE1* antisense transcripts. Sense or antisense (McElver *et al.*, 2001) read-through transcription of flanking genes from the same type of T-DNA-associated promoters has been reported (Ülker et al., 2008). For instance, the terminator used for pGKB5 (Bouchez *et al.*, 1993) of the FLAG lines is weak, and in conjunction with the 35S promoter can lead to read-through transcription (Meierhoff *et al.*, 2003; Yoo *et al.*, 2005; Ülker *et al.*, 2008). The 10-fold difference in the antisense transcript abundance in *the1-3* versus *the1-4* is probably due to the strong activity of the bidirectional MAS promoter (*the1-3*) and the weak read-through of the 35S promoter in the FLAG line, *the1-4*. The high levels of antisense transcripts in *the1-3* might act similarly to natural antisense transcripts (NATs) at different levels including mRNA processing, cellular transport, and translation (Faghihi and Wahlestedt, 2009; Britto-Kido *et al.*, 2013).

The findings of Guo *et al.* (2009*a*, *b*) that *herk1* and *herk2* only in combination with the hypermorphic *the1-4* allele inhibit cell elongation might indicate cell wall integrity defects in these mutants. Another possibility would be that HERK1 and HERK2 act antagonistically to THE1. Seemingly opposite functions of CrRLK family members have been observed for FER and ANX1/2 during pollination (Escobar-Restrepo *et al.*, 2007; Boisson-Dernier *et al.*, 2009; Miyazaki *et al.*, 2009). Kessler *et al.* 2015) found that FER and ANX most probably do not compete for the same ligand and proposed that an as yet unknown mechanism leads to antagonistic functions. Moreover, it is not formally excluded that the two *HERK* mutants used by Guo *et al.* (2009*a*, *b*) are hypermorphic alleles, since the authors did not report on expression analyses of the 5' end of these mutants, and the insertion of the T-DNA would allow full ECD expression in both of them.

Our results illustrate one of the pitfalls of using T-DNA insertion lines to infer gene function from quickly characterized mutants and in particular mutants for RLKs. Finally, the availability of both gain-of-function and loss-of-function alleles for *THE1* will be helpful for the dissection of cell wall integrity signaling networks in the future.

## Supplementary data

Supplementary data are available at *JXB* online.

Table S1. List of primers used.

Fig. S1. Root growth of *the1-3*, *the1-4*, and THE1:GFP seedlings in cellulose-deficient genetic backgrounds

Fig. S2. Ectopic lignification of etiolated seedlings of *the1* alleles in cellulose-deficient genetic backgrounds

Fig. S3. Inflorescence phenotypes of loss- and gain-of-function alleles in combination with cellulose-deficient backgrounds

Fig. S4. Semi-dominance of *the1-3* in combination with *cesa6*^*prc1-8*^.

## Supplementary Material

supplementary_table_S1_figures_S1_S4

## Abbreviations:

CaMV: *Cauliflower mosaic virus*
EMS: ethyl methanesulfonate
GFP: green fluorescent protein
MAS: mannopine synthase
MMLV: Moloney murine leukemia virus
qPCR: quantitative PCR
RLK: receptor-like-kinases
ROS: reactive oxygen species
RT–qPCR: reverse transcription–qPCR.
