# Supplementary material for: T-DNA alleles of the receptor kinase THESEUS1 with opposing effects on cell wall integrity signaling
Source: J Exp Bot. 2017 Aug 1;68(16):4583–93. doi: 10.1093/jxb/erx263 (PMC5853656; doi:10.1093/jxb/erx263)
Supplement: supplementary_table_S1_figures_S1_S4 [file erx263_suppl_supplementary_table_s1_figures_s1_s4.pdf]

SUPPLEMENTARY INFORMATION

T-DNA alleles of the receptor kinase THESEUS1 with opposing effects on cell wall integrity signaling

David Merz, Julia Richter, Martine Gonneau, Clara Sanchez-Rodriguez, Tobias Eder, Rodnay Sormani, Marjolaine Martin, Kian Hématy, Herman Höfte, Marie-Theres Hauser

Table S1 List of Primers used

**Name**	**Sequence**	**Specificity**
GFP-F	ACATGAAGCAGCACGACTTC	*GFP* construct
GFP-R	TGTAGTTGTACTCCAGCTTGT	*GFP* construct
THE1-Start	ATGGTGTTCACAAAATCATTAC	ECD-TM^{THE1}-YFP construct
TM-JTM-Lo	GAACTTATTAGTAGCGTCCAT	ECD-TM^{THE1}-YFP construct
THE1_3endF	AAGGTGAACCCTGCCTCGT	expression downstream T-DNA insertion
THE1_3endR	ATTGGAATCCCTGGAATGTGG	expression downstream T-DNA insertion
THE1_5endF	GTGGTCTTTGTCAACGCTAT	expression upstream T-DNA insertion
THE1_5endR	CATCATTATCCCATTGTCTCC	expression upstream T-DNA insertion
THE1_midF	GCTGATATCACTAATGCGAC	upstream T-DNA insertions, *the1-3, the1-4* genotyping, expression, antisense cDNA
THE1_midR2	CAGCAATCAGCAGAATCAATG	upstream T-DNA insertions
LB4	CGTGTGCCAGGTGCCCACGGAATAGT	*the1-3* genotyping; T-DNA specific expression
LB3	CTGAATTTCATAACCAATCTCGATACAC	*the1-4* genotyping; T-DNA specific expression
TUB9-F	GTACCTTGAAGCTTGCTAATCCTA	reference gene qPCR
TUB9-R	GTTCTGGACGTTCATCATCTGTTC	reference gene qPCR
UBQ-F	CTCCTTCTTTCTGGTGGTAAACGT	reference gene qPCR
UBQ-R	AACCCTTGAGGTTGAATCATCC	reference gene qPCR
5g19110/EDGP-F	CTACAATGCTCTTGCTCAGTC	*THE1* target gene, qPCR
5g19110/EDGP-R	ACCGTCGATAAACGCCAAAC	*THE1* target gene, qPCR
2g26530/AR781-F	CATCAGCTTGCGTGTCTTGC	*THE1* target gene, qPCR
2g26530/AR781-R	CCCTCTTCCTCGTGAACTCG	*THE1* target gene, qPCR

Supplementary Figures

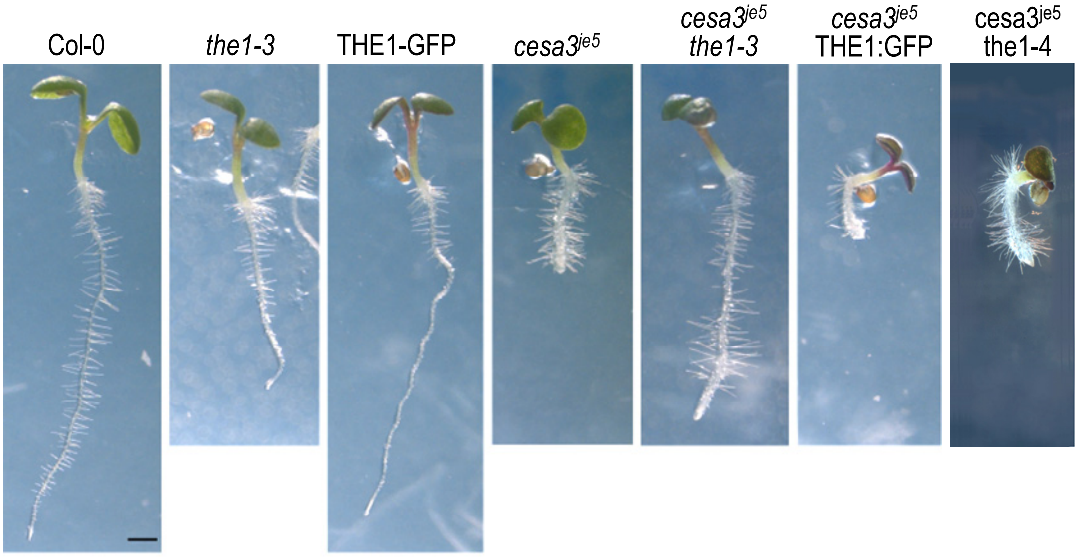

Figure S1 Root growth of *the1-3*, *the1-4* and 35S::THE1:GFP seedlings in cellulose deficient genetic backgrounds. Black bar corresponds to 1 mm.

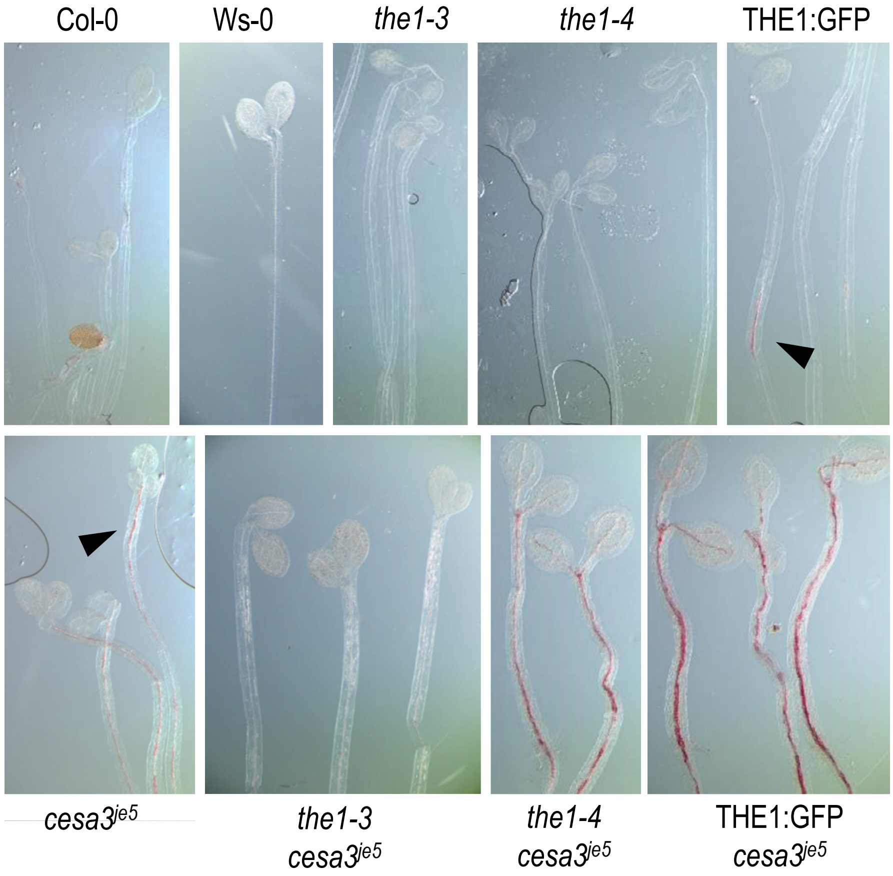

Figure S2 Ectopic lignification of etiolated seedlings of *the1* alleles in cellulose deficient genetic backgrounds.

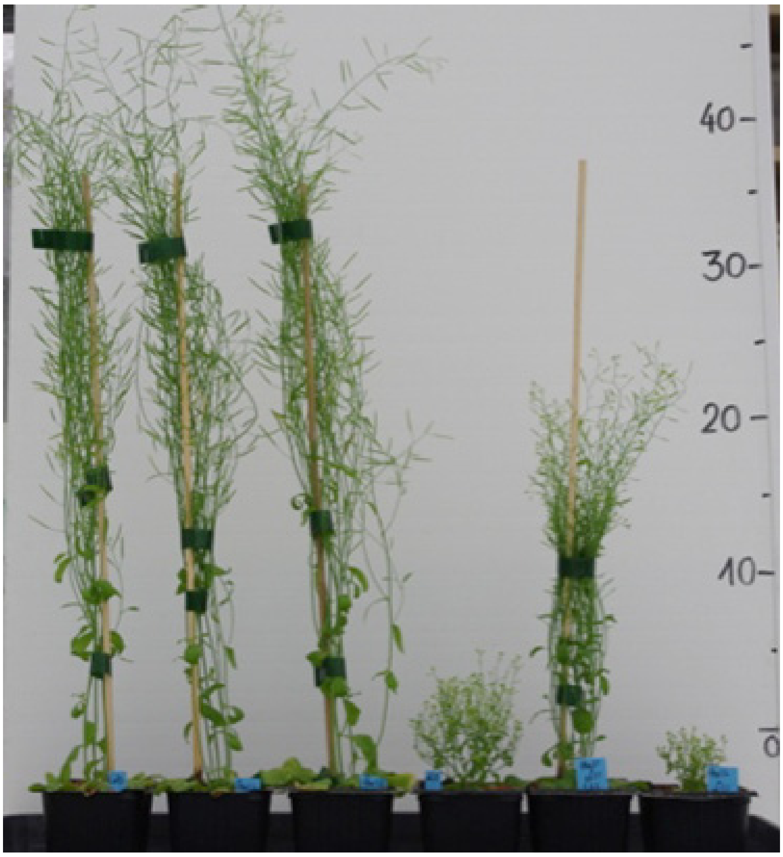

Figure S3 Inflorescence phenotypes of loss- and gain-of-function alleles in combination with cellulose deficient backgrounds.

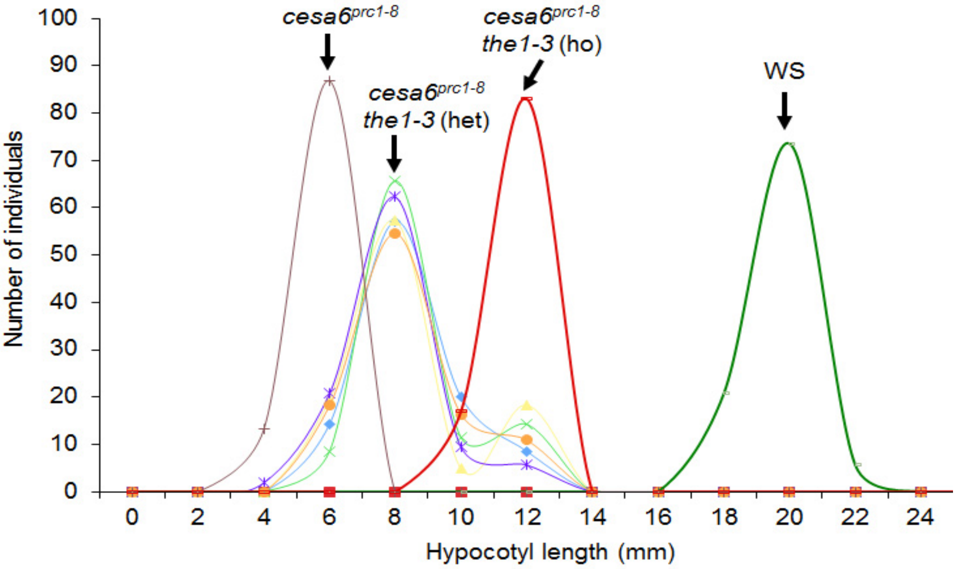

Figure S4 Semidominance of *the1-3* in combination with *cesa6*$^{prc1\text{-}8}$. Seedlings were cultivated on MS medium without sucrose for 7 days in the dark. Data represent frequency distributions of hypocotyl lengths. All seedlings except wild type (WS) were homozygous for *cesa6*$^{prc1\text{-}8}$ and either wild type, heterozygous (het) or homozygous (ho) for *the1-3*.
